# Expression of GSK-3β in renal allograft tissue and its significance in pathogenesis of chronic allograft dysfunction

**DOI:** 10.1186/1746-1596-7-5

**Published:** 2012-01-13

**Authors:** Qiang Yan, Baoyao Wang, Weiguo Sui, Guimian Zou, Huaizhou Chen, Shenping Xie, Hequn Zou

**Affiliations:** 1 Kidney Tranplantation and Dialysis Center of PLA NO.181 Hospital, Clinical College of Southern Medical University, Guilin 524001, Guangxi, P.R.China; 2 Southern Medical University, Guangzhou 510630, Guangdong, P.R.China; 3 Institute of Urology and Nephrology, The Third Affiliated Hospital of Southern Medical University, Guangzhou 510630, China

**Keywords:** kidney transplantation, GSK-3β, inflammatory cell infiltration

## Abstract

**Objective:**

To explore the expression of Glycogen synthase kinase 3 beta (GSK-3β) in renal allograft tissue and its significance in the pathogenesis of chronic allograft dysfunction.

**Methods:**

Renal allograft biopsy was performed in all of the renal allograft recipients with proteinuria or increased serum creatinine level who came into our hospital from January 2007 to December 2009. Among them 28 cases was diagnosed as chronic allograft dysfunction based on pahtological observation, including 21 males with a mean age of 45 ± 10 years old and 7 females with a mean age of 42 ± 9 years old. The time from kidney transplantation to biopsy were 1-9 (3.5) years. Their serum creatinine level were 206 ± 122 umol/L. Immunohistochemical assay and computer-assisted genuine color image analysis system (imagepro-plus 6.0) were used to detect the expression of GSK-3β in the renal allografts of 28 cases of recipients with chronic allograft dysfunction. Mean area and mean integrated optical density of GSK-3β expression were calculated. The relationship between expression level of GSK-3β and either the grade of inflammatory cell infiltration or interstitial fibrosis/tubular atrophy in renal allograft was analyzed. Five specimens of healthy renal tissue were used as controls.

**Results:**

The expression level of the GSK-3β was significantly increased in the renal allograft tissue of recipients with chronic allograft dysfunction, compared to normal renal tissues, and GSK-3β expression became stronger along with the increasing of the grade of either inflammatory cell infiltration or interstitial fibrosis/tubular atrophy in renal allograft tissue.

**Conclusion:**

There might be a positive correlation between either inflammatory cell infiltration or interstitial fibrosis/tubular atrophy and high GSK-3β expression in renal allograft tissue.

**Virtual slides:**

The virtual slide(s) for this article can be found here:

http://www.diagnosticpathology.diagnomx.eu/vs/9924478946162998.

## Introduction

Kidney transplantation is the optimal renal replacement therapy for the patients with end stage renal disease (ESRD). As the application of more new effective immunodepressants and the development of transplant technique, the incidence of acute rejection is obviously decreasing in the early stage post transplantation, but the long term survival of renal allografts is still a challenge of clinical medicine. Recent researches have found that chronic renal allograft dysfunction is the main factor influence on the long term survival of renal allograft. The main clinical manifestations of chronic renal allograft dysfunction are serum level of creatinine is slowly creeping upward company with proteinuria and hypertension, and progression into ESRD need kidney transplantation again or on maintenance dialysis. The main pathogenic course of chronic renal allograft dysfunction is glomerulosclerosis, vasculopathy, atrophy of tubule and chronic renal interstitial fibrosis. All the pathogenic changes are associated with lymphocyte, plasmocyte and mastocyte infiltration in the renal tissue. Researches have proved that chronic inflammatory was the key pathogenic course of nephron loss in various of kidney disease, including chronic renal allograft dysfunction. Recently, researches discovered that GSK-3β mediated chronic inflammatory related to deterioration of renal allograft function, but the mechanism has not been fully interpreted. In this study, we mainly detected the expression of GSK-3β in the tissue of renal allograft, and analyzed the relationship between the expression of GSK-3β and inflammatory cell infiltration in renal interstitium, interstitial fibrosis and tubule atrophy. The role of GSK-3β in chronic allograft dysfunction was discussed.

## Methods

### Clinical data

The renal biopsy samples were collected from kidney transplant patients with proteinuria and elevated serum creatine from January, 2007 to December 2009 in Guilin No.181 hospital. In the 28 cases consistented with clinical diagnosis of chronic allograft dysfunction, 21 cases were males (age 45 ± 10 years) and 7 cases were females (age 42 ± 9 years). The durations after kidney transplantation were 1~9 years (mean 3.5 years) and the mean levels of serum creatine were 206 ± 122 μmol/L. The triple immunosuppressant protocols were cyclosporine + mycophenolate mofetil + prednisone in 18 patients and tacrolimus + mycophenolate mofetil + prednisone in 9 patients and sirolimus +.mycophenolate mofetil + prednisone in 1 patient. Before renal biopsy, color Doppler supersonic detection in renal allograft and serum drug levels were performed to exclude acute rejection, nephrotoxicity of immunosuppressant, obstruction/backstreaming of ureter, thrombosis or embolism in renal arteries or veins and other diseases. The donors and recipients were matching in ABO blood type, and two or more matching in HLA, and the results of lymphocytotoxicity test were less than 10%, and the results of panel reaction antibody (PRA) were negative. The renal samples of 5 cases of control were collected from routine donor renal biopsy before transplantation, and the pathologic manifestation of which was normal. Informed consents were obtained from all patients that participated to the study. This study was performed under the supervision of Institutional Review Board of Southern Medical University, and abided the Helsinki Declaration on ethical principles for medical research involving human subjects.

### Histological examination

The paraffin-embedded kidney sections were incised into 3-μm-thick tissue sections that were deparafinized through xylene and hydrated through graded ethanol (100%, 96%, 90%, and 70%) and distilled water. The sections were stained by standard histology procedures, including hematoxylin/feosin stain, periodic-acid schiff (PAS) stain, masson trichrome and periodic schiff- methenamine (PASM) stain. The slices were observed under microscope in a blind manner, including inflammatory cell infiltration in renal tissues, increased mesangial matrix and extracellular matric, proliferation of mesangial cells, epithelium and endothelium, adhesions and sclerosis, thickening of glomerular basement membranes and double track sign; thickening of peritubular capillaries basement membrane, interstitial fibrosis, inflammatory cell infiltration, tubular atrophy and intimal thickening of artery.

### Pathologic diagnosis

Diagnosis criteria of chronic allograft dysfunction: thickening and splitting appearance of glomerular basement membrane, thickening of basement membrane of peritubular capillaries, and/or diffuse monocyte infiltration of mononuclearcell, interstitial fibrosis/diffuse tubular atrophy, and/or thickening of artery intima, excluding acute rejection, nephrotoxicity of immunosuppressant, obstruction/back- streaming of ureter and other diseases.

The degree of inflammatory cell infiltration in tubulointerstitial was described as mild, midrange and severe. There were few and scatter inflammatory cell infiltration in 10-25% of parenchyma was defined as mild; inflammatory cell infiltration in 26-50% of parenchyma was defined as midrange; inflammatory cell infiltration in > 50% of parenchyma was defined as serve [1].

Degree of interstitial ﬁbrosis and tubular atrophy: IF/TA-I, as mild interstitial ﬁbrosis and tubular atrophy (< 25% of cortical area); IF/TA-II, as moderate interstitial ﬁbrosis and tubular atrophy (26-50% of cortical area); IF/TA-III, as Severe interstitial ﬁbrosis and tubular atrophy/loss (> 50% of cortical area) [1].

### Immunohistochemisty examination

Immunohistochemisty with EnVision was used to detect the expression of GSK-3β protein in the renal allograft tissue. 3-μm-thick tissue sections were hydrated through graded ethanol, and endogenous peroxidase was blocked with 3% peroxide. GSK-3β was repaired with microwave for 15 minute. We incubated primary antibody (rabbit anti-human GSK polyclonal antibody, 1:100, Wuhan Boster Biological Technology, Ltd) to detect GSK-3β at overnight at 4°C. After washing with PBS, we incubated the tissue sections with the second antibody (mice anti-rabbit monoclonal antibody, Maixin Biological Technology Development Co. Fujian) for 30 min at 37°C, and then washing with PBS, DAB coloration and re-stained with hematoxylin.

Immunohistochemisty test of IgG, IgA, IgM, Clq, C4c and C4d were performed in the same way.

### Image analysis

Images were acquired using a Leica DMR-X microscope coupled to a Leica DC500 digital camera (Leica, Wetzlar, Germany), using the image analysis system Quantimet Q550 (Leica Imaging Systems). Ten randomly selected discontinuous fields (400 x) per kidney were evaluated, including tubulointerstitial in renal cortex, medulla and the conjunction region, (but not including glomulular and vessels). More than 60 tubulars in each biopsy section. The positive area was yellowly stained and Image-Pro Plus software was used to quantify the integrated optical density. The ratio of positive area to total tubulointerstitial area (not including the area of tubular lumina) presented the relative amount of the substance expression in tubuloinstitial.

### Statistic analysis

Results are expressed as means ± SD. Statistical analysis was performed using SPSS 13.0 (SPSS, Chicago, IL). Measurement data was analyzed with one way ANOVA, and the relationship was analyzed with linear correlation (Pearson). Statistic significance was set at P < 0.05 level.

## Results

### Histological manifestation

The main histological manifestation of renal allograft in the 28 cases of chronic renal allograft dysfunction as: interstitial fibrosis and tubular atrophy accompany with infiltration of inflammatory cells including lymphocyte and monocyte, enlarge of some tubular lumens, thickening of glomerular basement membrane as double trace sign and focal segmental glomerulosclerosis, thickening of artery intimae. Discrepancy to the presentation of acute rejection, nephrotoxicity of immunosuppressant and obstruction uropathy/reflux nephropathy.

### The expression of GSK-3β in renal tissue

**Figure 1:**
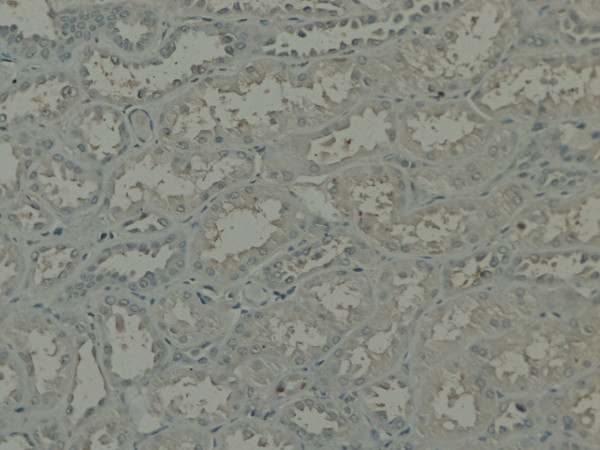
**GSK-3β expression in renal tissue with different degree of inflammatory infiltration**: control group.

**Figure 2:**
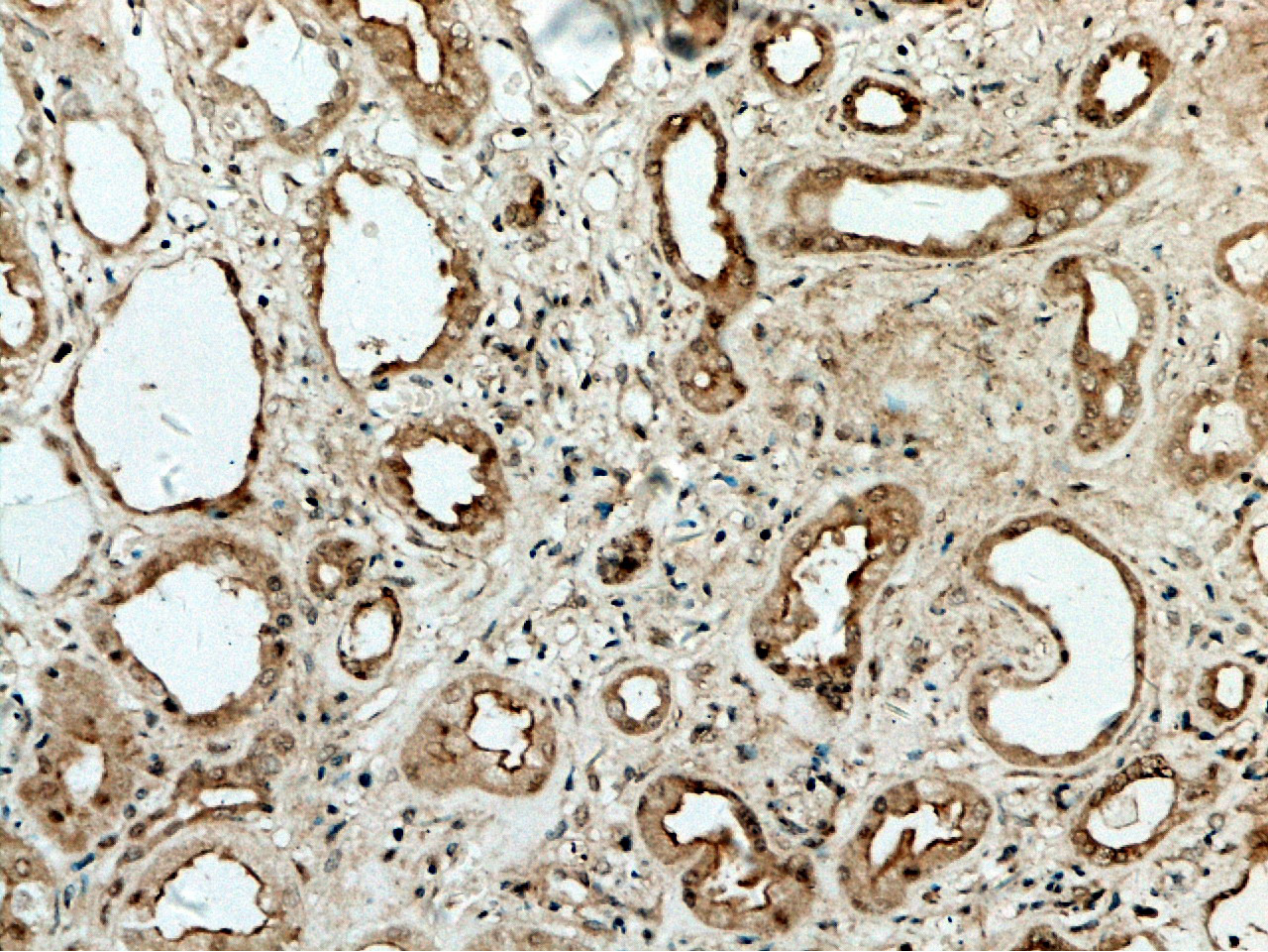
**GSK-3β expression in renal tissue with different degree of inflammatory infiltration**: mild degree of inflammatory infiltration.

**Figure 3:**
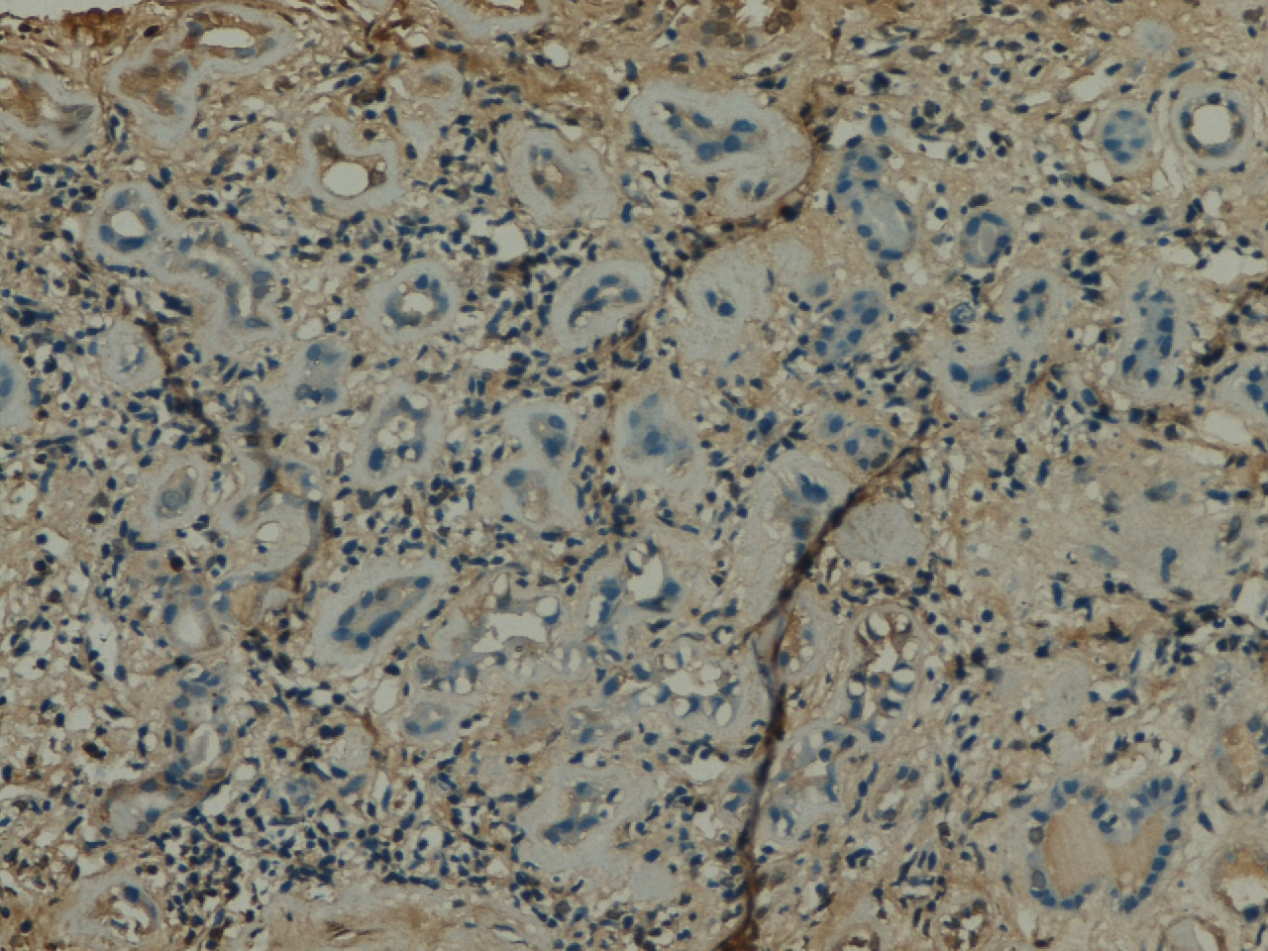
**GSK-3β expression in renal tissue with different degree of inflammatory infiltration**: moderate inflammatory infiltration.

**Figure 4:**
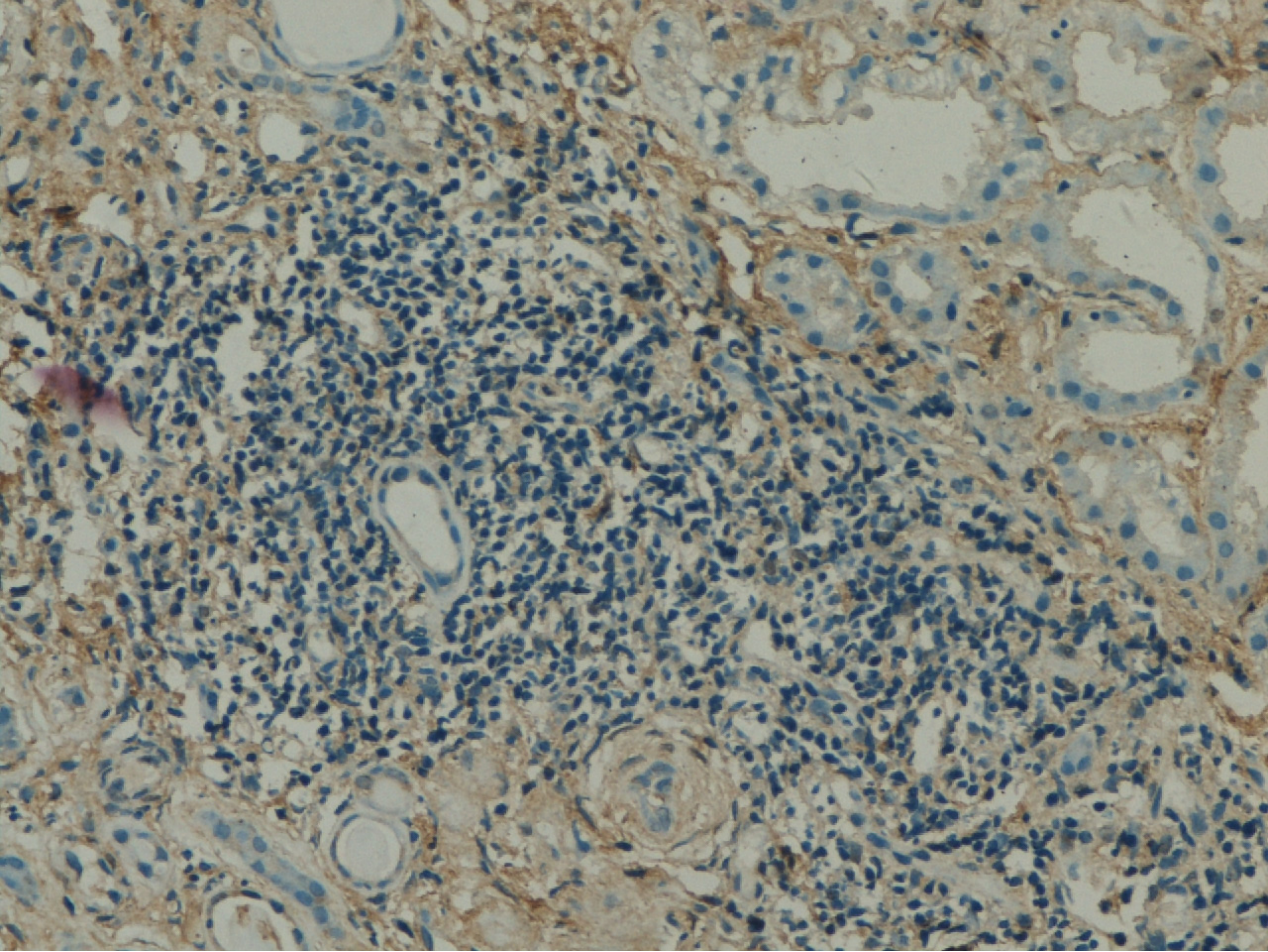
**GSK-3β expression in renal tissue with different degree of inflammatory infiltration**: severe inflammatory infiltration.

**Figure 5:**
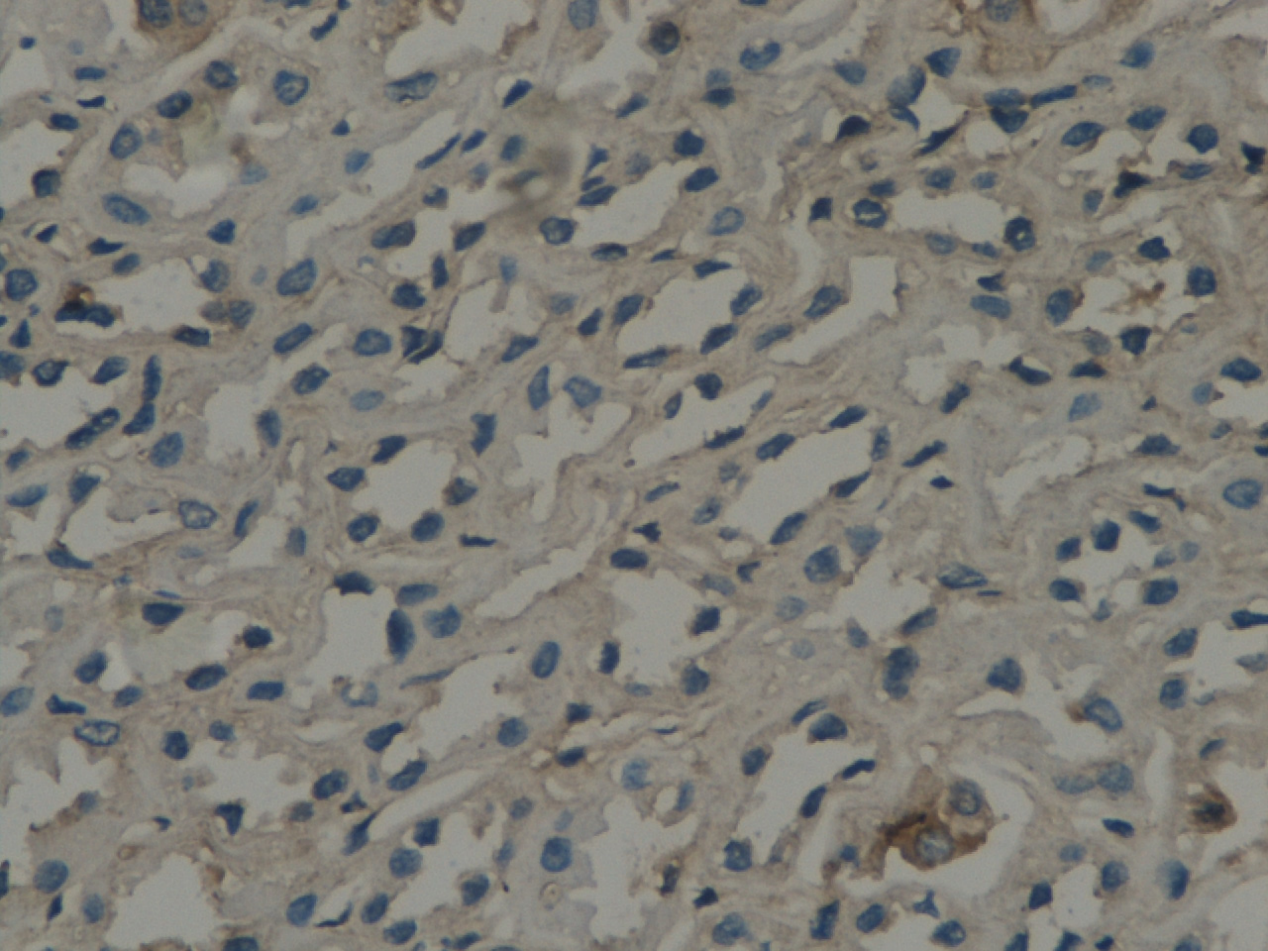
**GSK-3β expressions in renal tissue with different degree of interstitial fibrosis/tubular atrophy**: control group.

**Figure 6:**
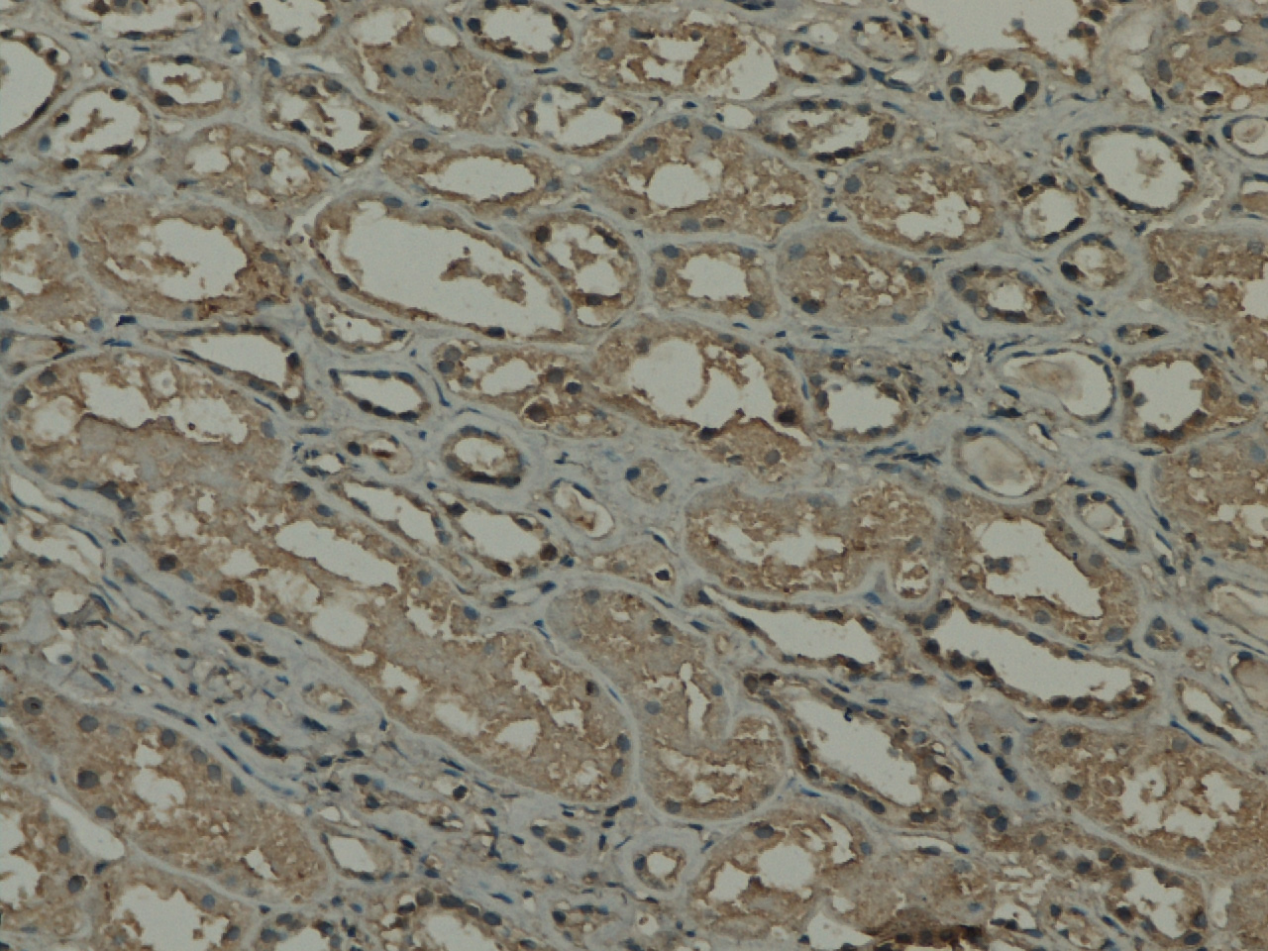
**GSK -3β expressions in renal tissue with different degree of interstitial fibrosis/tubular atrophy**: IF/TA-I Group.

**Figure 7:**
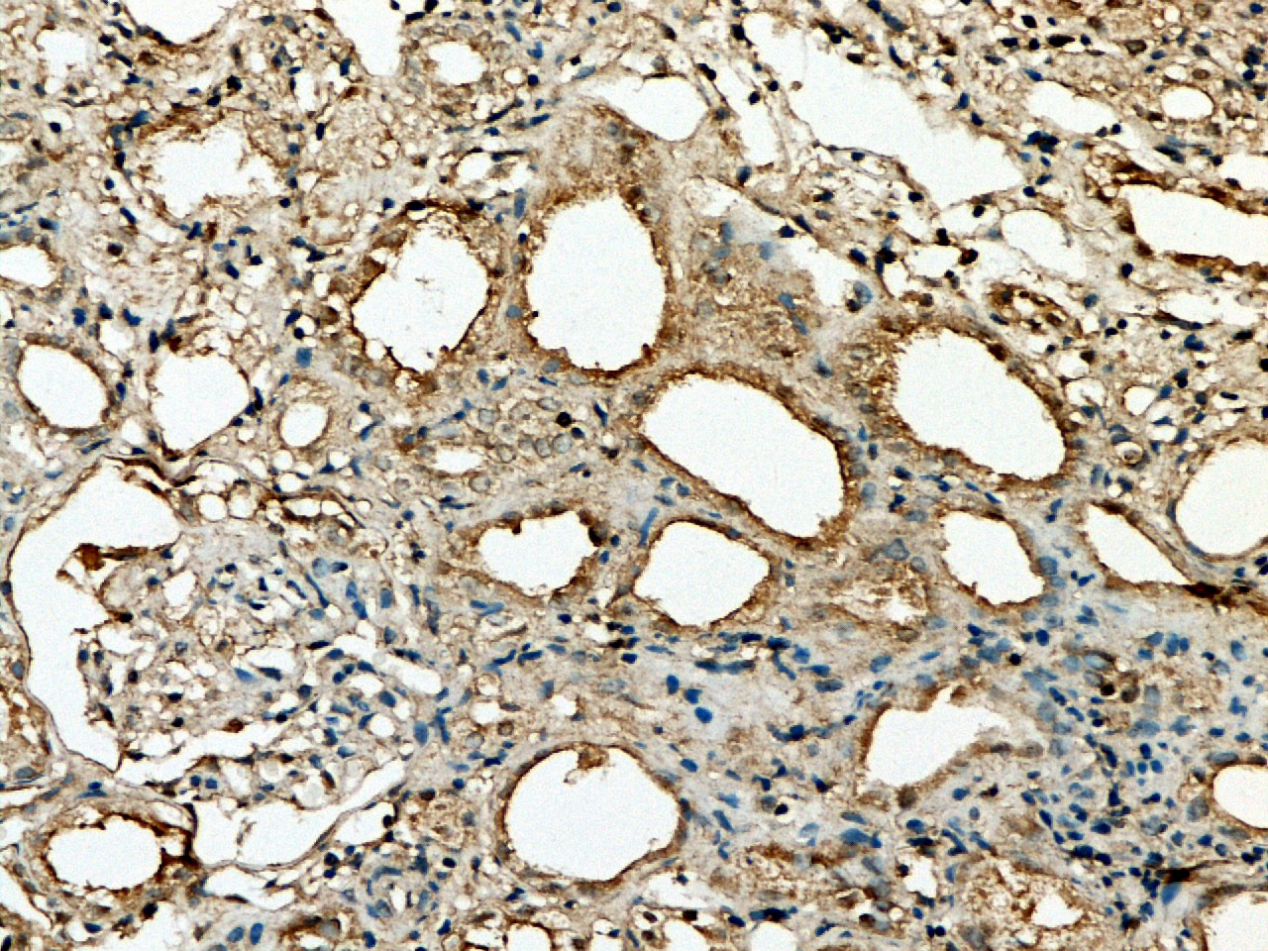
**GSK -3β expressions in renal tissue with different degree of interstitial fibrosis/tubular atrophy**: IF/TA-II Group.

**Figure 8:**
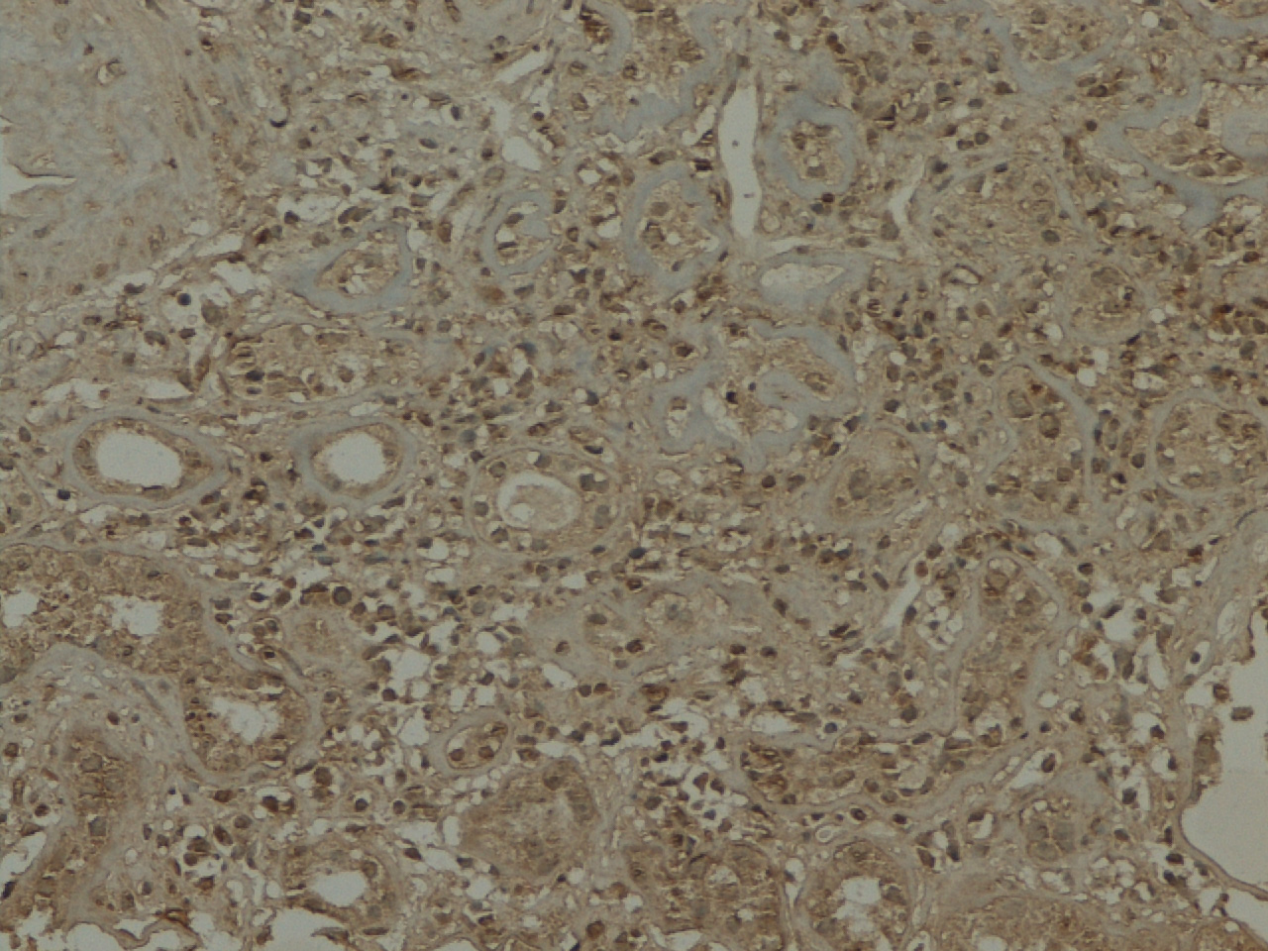
**GSK -3β expressions in renal tissue with different degree of interstitial fibrosis/tubular atrophy**: IF/TA-III Group.

There was weak expression of GSK-3β in the cytoplasm of tubular epithelia cell in normal renal tissue. However, in the dysfunction renal allograft tissue, there were strong cytoplasm GSK-3β expression in tubular epithelia cell, and stronger expression in the patients with more severity tubulointerstitial damage. (Shown in table 1, table 2 and Figure 1, 2, 3, 4, Figure 5, 6, 7, 8).

**Table 1:** The area and integrated optical density of GSK-3β expression in renal tissue with different degree of inflammatory infiltration

	n	Area of GSK expression(%)	GSK(IOD)
Control Group	5	17.45 ± 19.63	21757 ± 23765
Mild Group	11	33.15 ± 9.16^**⋇**^	48331 ± 20335^**⋇**^
Moderate Group	10	45.77 ± 14.56^**⋇⋆**^	70702 ± 24891^**⋇⋆**^
Severe Group	7	60.83 ± 13.53^**⋇⋆♦**^	96300 ± 28169^**⋇⋆♦**^

^⋇^: p < 0.05, vs Control Group; ^⋆^: p < 0.05, vs Mild Group; ^♦^: p < 0.05, vs Moderate Group.

GSK: glycogen synthase kinase; IOD: integrated optical density.

**Table 2:** The area and integrated optical density of GSK-3β expression in renal tissue with different degree of interstitial fibrosis and tubular atrophy

	n	GSK(%)	GSK(IOD)
Control group	5	17.45 ± 19.63	21757 ± 23765
IF/TA-I group	12	35.48 ± 11.90^**⋇**^	52017 ± 23216^**⋇**^
IF/TA-II group	11	48.50 ± 16.51^**⋇⋆**^	741235 ± 26216^**⋇**^
IF/TA-III group	5	57.80 ± 14.40^**⋇⋆**^	94615 ± 33855^**⋇⋆**^

^⋇^: p < 0.05, vs Control Group; ^⋆^: p < 0.05, vs IF/TA-I Group; ^♦^: p < 0.05, vs IF/TA-II Group.

GSK: glycogen synthase kinase; IOD: integrated optical density.

### The relationship of GSK-3β expression and inflammatory infiltration, interstitial fibrosis and tubular atrophy

**Figure 9:**
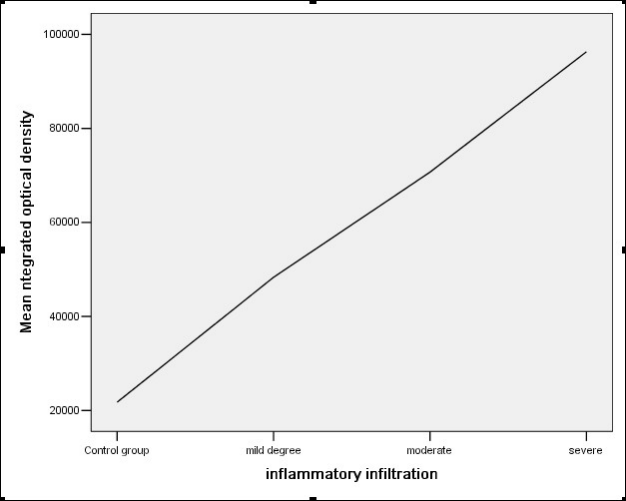
**The correlation between GSK-3β expression and inflammatory infiltration (r = 0.688, p < 0.001)**.

**Figure 10:**
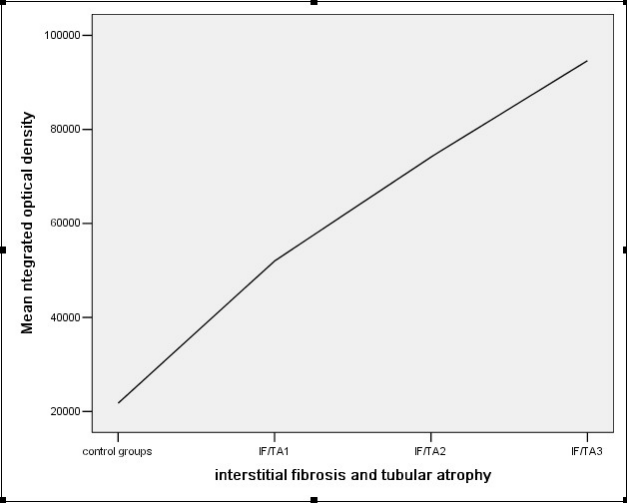
**The correlation between GSK-3β expression and interstitial fibrosis and tubular atrophy (r = 0.584, p < 0.001)**.

In the renal tissue of chronic renal allograft dysfunction, the integrated optical density (IOD) was higher than normal renal tissue by analysis with Image-Pro software, and the highest of IOD in more severity of inflammatory infiltration, interstitial fibrosis and tubular atrophy(Figure 9,10).

## Discussion

Renal allograft chronic dysfunction is the main factor influence on the long term survival of renal allograft, and is due to multiple causes. The clinical manifestations are gradually loss of renal allograft function and serum creatinine slowly creeping upward. The risk factors of renal allograft dysfunction include immune factors, such as times of early acute rejection and matching of HLA and protocol of immunosuppression, and non-immune factors such as ischemia/reperfusion injury, delayed graft function, nephrotoxicity of calcineurin inhibitors, hypertension and dyslipidemia, and progressively develop into end stage renal disease. Chronic renal allograft dysfunction morphological appearance as thickening of glomerular basement membrane as double contours sign, thickening of the basement membrane of peritubular capillary, and/or diffuse mononuclear cell infiltration and fibrosis in interstitium and difusse tubular atrophy, and/or thickening of artery intima.

Our study has found that GSK-3β was weakly expressed in normal renal tissue, and was strongly expressed in chronic dysfunction renal allograft tissue, and was stronger in the more severity of pathological changes. The IOD of GSK-3β was higher than that in normal renal tissue, and increasing with the severity of inflammatory infiltration, interstitial fibrosis and tubular atrophy, which suggest that the correlation of GSK-3β expression and interstitial inflammatory infiltration, interstitial fibrosis/tubular atrophy. It was necessary to further study the pathogenic mechanism of GSK-3β in the course of interstitial inflammatory infiltration, interstitial fibrosis and tubular atrophy.

Recent researches have found that the GSK-3β family of serine/threonine kinases extensively existed in the eucaryon, and mainly express in the lung, kidney and brain, and influence on construction, growth, activation and apoptosis of the cells by involving the basic cellular processes such as signal transduction, regulation of insulin, gene transcription and translation. The activation of GSK-3β mainly through phosphorylation of serine-9 near the N-terminus, and is associated to the regulation of signal pathway of Wnt, protein kinase B/Akt and NF-κB. The abnormal activatin of GSK-3β is associated with several kidney diseases, such as diabetic nephropathy, mesangioproliferative glomerulonephritis, lupus nephritis and chronic allograft nephropathy. Recently, several researches revealed that GSK-3β involved in human inflammatory diseases by controling the production of inflammatory factors and involvement into NF-κB signal pathway, and the activation of NF-κB is the center step of inflammatory reaction. Giannopoulou M. et al. found in cultured renal tubular epithelium that hepatocyte growth factor (HGF) has a potent suppressive effect on NFkappaB activation, which was mediated by GSK-3β. HGF can inhibit renal inflammation and proinflammatory chemokine expression by disrupting NF-kB signaling [2]. Adminis- tration of a GSK-3 inhibitor potently suppressed the proinflammatory response in mice receiving lipopolysaccharide and mediated protection from endotoxin shock [3]. Study of Gong and colleagues had proved that GSK-3β was a new marker of chronic renal allograft dysfunction and mediating proinflammatory NF-kB activation and renal inflammation. GSK-3β over expression in the damaged tubular epithelial cells and the magnitude of GSK-3β phosphorylation (p-GSK, inactivation state) was inversely correlated with the degree of injury as assessed by Banff criteria. The higher level of p-GSK expression, the lower level of tubular atrophy and interstitial fibrosis [4].

Chronic renal allograft dysfunction is an important factor influence on the long term survival of transplant kidney, but the pathogenic mechanism is still not fully interpreted. Infiltration of mononuclear cell (monocyte and lymphocyte) often presents in the renal tissue of chronic dysfunction and relate to endarteritis and interstitial fibrosis. Impairment of renal tubular epithelium and interstitial inflammatory infiltration play an important role in various kidney diseases. Inflammatory factors induce secretion of extracellular matrix by interstitial fibrocyte and result in interstitial fibrosis. In vitro and in vivo experiments shown that GSK-3β involved in inflammatory diseases and inhibition of GSK-3β retarded the progression of these diseases. In the diabetic nephropathy mice experiment model, troglitazone a peroxisome proliferator -activated receptor gamma (PPAR gamma) agonists, ameliorate renal fibrotic lesions through reverse high glucose induced expressions of GSK-3β and inhibition epithelial-mesenchymal transition [5]. In the rats with experimental mesangial proliferative glomerulonephritis, inhibitors of cyclin- dependent kinase (CDK)/GSK-3β were the effective antagonists of proliferating and immunity renal disease [6]. CDK/GSK-3β inhibitors suppress pathogenic proliferation, apoptosis, and inflammation, and promote regeneration of injured tissue in parenchymal renal diseases [7]. Sinha D and his colleague had studied the proximal tubular cells cultured in the absence of growth factors, found that GSK-3β inhibitor might improve the survival of proximal tubular cells. The main mechanism was by activation of Wnt signaling, which promoted the repair of damaged renal parenchyma [8].

Summary, there is intensive expression of GSK-3β in the renal tissue of the patients with chronic allograft dysfunction, and is associated with inflammatory cell infiltration of interstitium and interstitial fibrosis/tubular atrophy. Further studies are needed to investigate the pathogenic mechanism of GSK-3β expression in the renal tissue of chronic allograft dysfunction. Inhibition the activity of GSK-3β may be a new treatment target of prevention chronic renal allograft dysfunction.

## Competing interests

The authors declare that they have no competing interests.

## Authors' contributions

HZ and QY design the study, BW and GZ performed research and wrote the first draft of the manuscript, WS, HC and SX participated in the statistical analyses. All the authors read and approved the final manuscript.

## References

[B1] Solez K Colvin RB Racusen LC Banff 07 classification of renal allograft pathology: updates and future directions Am J Transplant 2008 8 4 753 760 10.1111/j.1600-6143.2008.02159.x 18294345

[B2] Giannopoulou M Dai C Tan X Hepatocyte growth factor exerts its anti- inflammatory action by disrupting nuclear factor-kappaB signaling Am J Pathol 2008 173 1 30 41 10.2353/ajpath.2008.070583 18502824 PMC2438283

[B3] Martin M Rehani K Jope RS Toll- like receptor-mediated cytokine production is differentially regulated by glycogen synthase kinase 3 Nat Immunol 2005 6 8 777 784 10.1038/ni1221 16007092 PMC1933525

[B4] Gong R Ge Y Chen S Glycogen synthase kinase 3beta: a novel marker and modulator of inflammatory injury in chronic renal allograft disease Am J Transplant 2008 8 9 1852 1863 10.1111/j.1600-6143.2008.02319.x 18786229

[B5] Lee YJ Han HJ Troglitazone ameliorates high glucose-induced EMT and dysfunction of SGLTs through PI3K/Akt, GSK-3{beta}, Snail1, and {beta}-catenin in renal proximal tubule cells Am J Physiol Renal Physiol 2009 10.1152/ajprenal.00475.2009 20015942

[B6] Pippin JW Qu Q Meijer L Direct in vivo inhibition of the nuclear cell cycle cascade in experimental mesangial proliferative glomerulonephritis with Roscovitine, a novel cyclin-dependent kinase antagonist J Clin Invest 1997 100 10 2512 2520 10.1172/JCI119793 9366565 PMC508451

[B7] Obligado SH Ibraghimov-Beskrovnaya O Zuk A CDK/GSK-3 inhibitors as therapeutic agents for parenchymal renal diseases Kidney Int 2008 73 6 684 690 10.1038/sj.ki.5002731 18094678

[B8] Sinha D Wang Z Ruchalski KL Lithium activates the Wnt and phosphatidylinositol 3-kinase Akt signaling pathways to promote cell survival in the absence of soluble survival factors Am J Physiol Renal Physiol 2005 288 4 F703 713 15572521 10.1152/ajprenal.00189.2004

